# Physical activity relates to adolescent social anxiety: evidence from a structural equation model involving resilience and self-efficacy

**DOI:** 10.3389/fpsyg.2026.1862222

**Published:** 2026-08-18

**Authors:** Ning Wang, Ye Chen, Mengmeng Li, Jifan Yang, Fangting Chen, Yijie Shi, Rujun Lv, Mariusz Lipowski

**Affiliations:** 1 Department of Physical Education, Lanzhou Institute of Technology, Lanzhou, Gansu, China; 2 School of Business, China West Normal University, Nanchong, China; 3 Chongqing Metropolitan College of Science and Technology, Chongqing, China; 4 Jinding First Primary School, Zhuhai High tech Zone, Zhuhai, China; 5 Jingyang Primary School, Zhuhai High tech Zone, Zhuhai, China; 6 Gdansk University of Physical Education and Sport, Gdansk, Poland; 7 Zhongshan University Affiliated Houhuan Primary School in the High-tech Zone of Zhuhai City, Zhuhai, China; 8 WSB Merito University in Gdansk, Gdansk, Poland

**Keywords:** adolescents, physical activity, resilience, self-efficacy, social anxiety

## Abstract

**Background:**

Physical activity is consistently associated with reduced anxiety in adolescents, yet the psychological mechanisms underlying this association remain unclear. This study examined whether resilience and self-efficacy independently and sequentially mediated the association between physical activity and social anxiety.

**Methods:**

A cross-sectional design was used with 986 Chinese adolescents. Physical activity, resilience, self-efficacy, and social anxiety were assessed using validated scales (PARS-3, CD-RISC, GSES, and SASC). Structural equation modeling was conducted in R, and indirect effects were tested using bias-corrected bootstrap procedures with 5,000 resamples.

**Results:**

Physical activity was significantly associated with lower social anxiety both directly (β = −0.420, *p* < 0.001) and indirectly. The indirect association through resilience was significant [β = −0.049, 95% CI [−0.064, −0.035]], whereas the indirect effect through self-efficacy and the serial mediation pathway were not significant. Absolute fit indices were acceptable (RMSEA = 0.064, SRMR = 0.073), but the incremental fit indices fell below conventional thresholds (CFI = 0.805, TLI = 0.796); the structural estimates should therefore be interpreted with corresponding caution.

**Conclusions:**

These findings are consistent with a model in which resilience functions as a proximal correlate linking physical activity to lower social anxiety, whereas general self-efficacy did not emerge as a significant mediator in this cross-sectional sample. Given the study design and the modest incremental fit of the model, these pathways should be read as theoretically plausible associations rather than causal effects. The results nonetheless underscore the potential value of fostering resilience within physical activity interventions, and they identify longitudinal designs and domain-specific efficacy measures as priorities for future research.

## 1 Introduction

Adolescent mental health has become an increasingly important public health concern worldwide, with anxiety emerging as one of the most prevalent and persistent psychological problems during this developmental period (Rapee et al., 2023). Its consequences are far from trivial: anxious adolescents show impaired academic functioning, strained peer relationships, reduced wellbeing, and an elevated risk of later psychopathology (de Lijster et al., 2018; Dickson et al., 2024). Among modifiable protective factors, physical activity has drawn particular attention, because higher activity levels are consistently associated with lower anxiety (Li et al., 2025; Ruiz-Ranz and Asín-Izquierdo, 2025). What remains unclear is how this association arises (Ligon et al., 2025). The present study therefore concentrates on two candidate psychological mechanisms—resilience and self-efficacy—and asks whether they account, separately or sequentially, for the association between physical activity and social anxiety in Chinese adolescents.

A substantial body of research has shown that physical activity is beneficial for mental health across the lifespan, and adolescents appear to be particularly sensitive to these effects (Li et al., 2023). Regular engagement has been linked to lower depression, stress, and anxiety, as well as better emotional regulation and subjective wellbeing (Lubans et al., 2016). Cognitive and behavioral dimensions of activity engagement appear to matter in their own right: among sports high school adolescents, cognitive-behavioral physical activity partially mediated the association between ruminative thinking and subjective wellbeing (Derelioglu et al., 2025), and evidence from Turkish samples indicates that perceived barriers and leisure-time patterns shape both activity engagement and perceived health (Kundakci et al., 2024). Direct evidence from Chinese adolescents points in the same direction. A four-wave longitudinal study of 1,259 students found that physical exercise predicted subsequent psychological resilience and, through it, lower social anxiety (Wu et al., 2025); a survey of 1,613 adolescents across 15 provinces likewise showed that exercise related to self-efficacy partly through resilience (Peng et al., 2025). Yet the magnitude of the activity–anxiety association has varied considerably across studies, which suggests that intervening psychological variables deserve closer scrutiny (Biddle et al., 2019; Xian et al., 2026).

One plausible mediator is resilience, which generally refers to the capacity to adapt positively in the face of adversity, stress, or challenge (Southwick et al., 2014). Resilience theory suggests that psychological resilience functions as a key protective resource that enables individuals to maintain or regain mental health under stressful conditions (Masten, 2018). From this perspective, physical activity may promote resilience by providing repeated opportunities for mastery, self-regulation, emotional release, and adaptive coping (Lubans et al., 2016). Participation in physical activity may also foster persistence, stress tolerance, and confidence in overcoming difficulties, all of which are central components of resilient functioning (Eime et al., 2013). Empirical studies have supported this view by showing that physically active adolescents tend to report higher resilience, and that resilience is associated with fewer internalizing symptoms, including anxiety (Luo et al., 2025). Adolescents with greater resilience are more likely to interpret stressors as manageable, employ adaptive coping strategies, and recover more effectively from negative emotional experiences (Fritz et al., 2018). Taken together, these findings suggest that resilience may serve as an important mediator linking physical activity to lower anxiety (Luo et al., 2025). Because the present data are cross-sectional, however, we treat this pathway as a theoretically plausible association rather than an established directional process; the longitudinal findings of Wu et al. (2025) make the proposed ordering credible, but they cannot settle it for the present sample.

Another relevant psychological mechanism is self-efficacy (Bandura, 1997). According to Bandura's social cognitive theory, self-efficacy refers to an individual's belief in their capacity to organize and execute the actions required to manage prospective situations (Bandura, 1997). Self-efficacy plays a central role in motivation, coping, emotional regulation, and behavioral persistence (Benight and Bandura, 2004). Adolescents with stronger self-efficacy beliefs are generally more likely to approach challenges with confidence, regulate stress effectively, and perceive themselves as capable of handling difficult situations, which may reduce anxiety (Muris, 2002). Physical activity may enhance self-efficacy by providing direct mastery experiences, which Bandura identified as the most influential source of efficacy beliefs (McAuley and Blissmer, 2000). Successfully engaging in exercise, achieving physical goals, and persisting through effortful activities may strengthen adolescents' perceptions of competence and control (McAuley et al., 2011). In turn, stronger self-efficacy may protect against anxiety by reducing feelings of helplessness, uncertainty, and perceived threat (Benight and Bandura, 2004). Existing evidence has shown that self-efficacy is negatively associated with anxiety and may mediate the relationship between health-related behaviors and psychological outcomes (Tang et al., 2022). These findings support the possibility of an indirect pathway from physical activity to anxiety through self-efficacy (Dishman et al., 2004). In the present study we deliberately assessed general rather than domain-specific self-efficacy, for three reasons. The Chinese version of the General Self-Efficacy Scale has well-documented psychometric properties in student samples (Wang et al., 2001); a general measure captures the cross-situational confidence that resilience theory treats as a transferable resource; and it preserves comparability with recent Chinese adolescent studies built on the same instrument (Leng et al., 2025). We acknowledge at the outset that exercise-specific or social self-efficacy lies conceptually closer to both the predictor and the outcome examined here—social cognitive theory itself characterizes efficacy beliefs as domain-bound (Bandura, 1997)—and this consideration proves important when interpreting the results.

Beyond their independent mediating roles, resilience and self-efficacy may also operate sequentially in a chain mediation process (Preacher and Hayes, 2008). This sequential pathway is theoretically meaningful because resilience and self-efficacy, although conceptually distinct, are closely related psychological resources (Masten, 2018). Resilience reflects adaptive recovery and psychological strength under adversity, whereas self-efficacy concerns perceived capability to cope with demands and challenges (Southwick et al., 2014). Resilience may facilitate the development of self-efficacy because adolescents who adapt effectively to stress and adversity are more likely to accumulate successful coping experiences, strengthen beliefs in their own competence, and develop a sense of control over future challenges (Benight and Bandura, 2004). In this sense, resilience may serve as an antecedent psychological resource that enhances self-efficacy (Luo et al., 2025). At the same time, greater self-efficacy may translate the adaptive potential of resilience into lower anxiety by promoting more confident coping, reducing threat appraisals, and enhancing emotional stability (Benight and Bandura, 2004). The stress-buffering model also supports this reasoning, as internal psychological resources may reduce the harmful effects of stress on mental health by improving coping capacity and perceived control (Cohen and Wills, 1985). Therefore, physical activity may first promote resilience, resilience may then enhance self-efficacy, and stronger self-efficacy may ultimately contribute to lower anxiety (Lubans et al., 2016). This sequential pathway captures a more refined mechanism than simple mediation because it reflects how one psychological resource may foster another, thereby transmitting the influence of physical activity to emotional outcomes (Preacher and Hayes, 2008). The ordering specified here—resilience preceding self-efficacy—is not the only defensible one. Some investigators model self-efficacy as the antecedent resource, arguing that confident individuals appraise stressors more benignly and thereby become more resilient (Leng et al., 2025), and reciprocal influence over time is at least as plausible developmentally. We adopted the resilience-first specification because recent evidence from Chinese adolescents indicates that resilience partially transmits the association between exercise and self-efficacy (Peng et al., 2025), and because mastery-based accounts treat accumulated adaptive experience as a principal source of efficacy beliefs (Benight and Bandura, 2004). Cross-sectional data cannot adjudicate among these alternatives; we therefore present the serial model as one theoretically motivated specification and return to its rivals in the Discussion.

Despite the growing literature on physical activity and adolescent mental health, several important gaps remain (Ruiz-Ranz and Asín-Izquierdo, 2025). What earlier work has established can be summarized briefly. Reviews and meta-analyses document a reliable inverse association between physical activity and anxiety in youth, although the psychological processes behind this link have received far less attention (Biddle et al., 2019; Li et al., 2025). Mediation studies have typically examined one resource at a time: resilience transmitted part of the exercise–social anxiety association in a longitudinal Chinese adolescent sample (Wu et al., 2025), while self-efficacy has mediated associations between activity and outcomes such as negative affect and intervention response (Dishman et al., 2004; Tang et al., 2022). Serial models combining the two resources do exist, but they have addressed different outcomes—academic stress, for example (Leng et al., 2025)—or different populations, such as college students assessed with sleep-related mediators (Li et al., 2024). To our knowledge, no study has tested resilience and self-efficacy as serial mediators between physical activity and social anxiety in Chinese adolescents. It therefore remains unclear whether activity relates to social anxiety through resilience alone, through self-efficacy alone, or through a chain in which resilience fosters efficacy beliefs; answering that question requires estimating all three pathways within a single model (Hayes, 2013).

Accordingly, the present study aimed to examine a serial mediation model in which physical activity was associated with social anxiety both directly and indirectly through resilience and self-efficacy among adolescents. Three theoretical perspectives motivated the model, and each supplies a distinct link in the hypothesized chain. Resilience theory anchors the first step: the mastery, self-regulation, and recovery experiences afforded by regular activity should strengthen adaptive capacity (Masten, 2018). Social cognitive theory anchors the second, treating accumulated adaptive experience as a wellspring of perceived capability (Bandura, 1997). The stress-buffering model frames the outcome side, regarding both resources as internal buffers that weaken the impact of stressors on emotional functioning (Cohen and Wills, 1985). Estimating the three perspectives jointly within one structural model makes it possible to compare separate and sequential indirect associations and to identify which links the data actually support (Preacher and Hayes, 2008). The results may also carry practical implications for school-based and health promotion programmes aimed at reducing adolescent social anxiety (Rodriguez-Ayllon et al., 2019).

**Figure 1:**
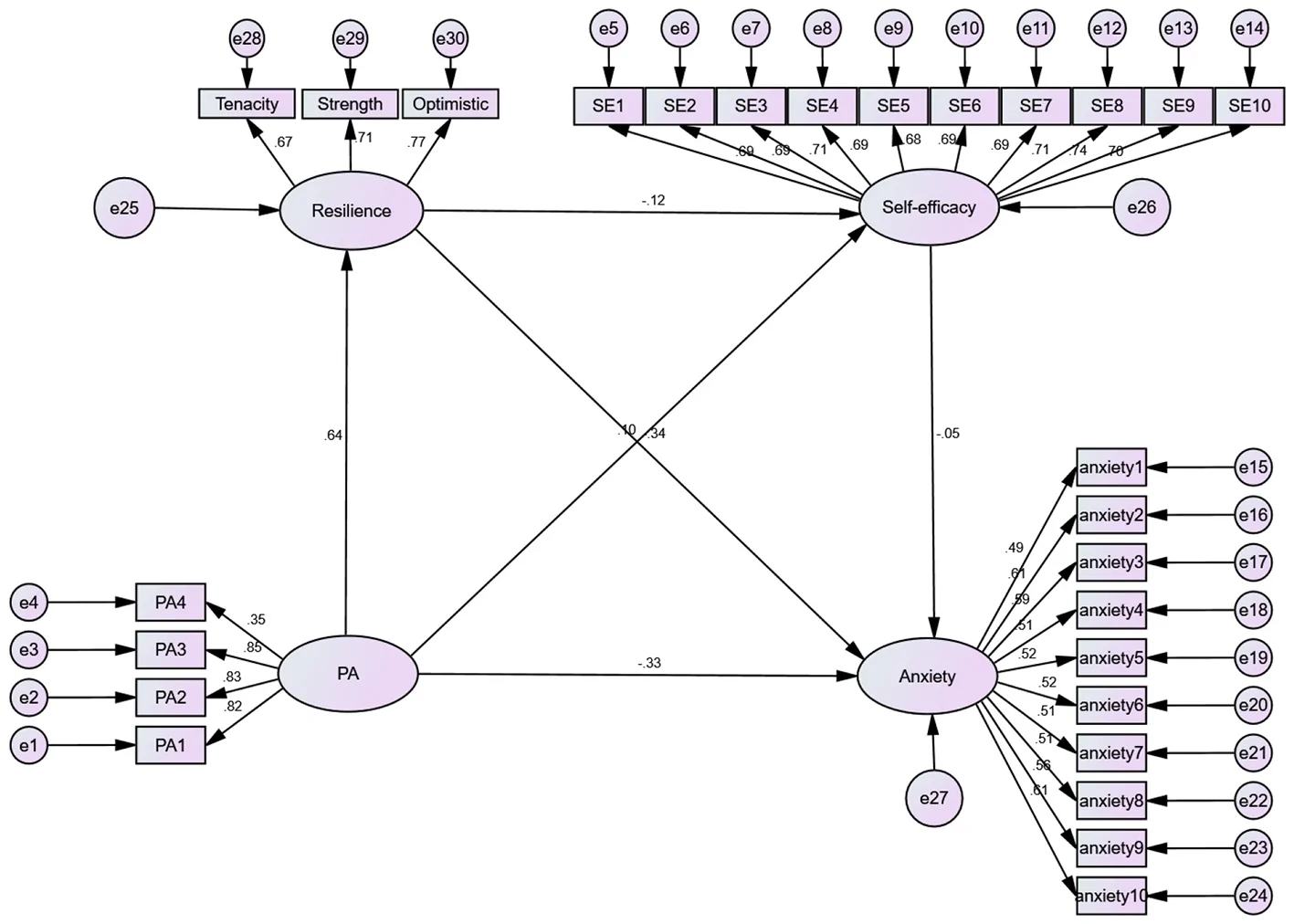
Conceptual model of research with standardized path coefficients.

Based on the theoretical and empirical considerations outlined above, we proposed that physical activity would be negatively associated with social anxiety in adolescents. (1) We further expected that resilience would mediate the association between physical activity and anxiety, such that greater physical activity would be associated with higher resilience, which in turn would be associated with lower anxiety. (2) In addition, we proposed that self-efficacy would mediate the association between physical activity and anxiety. (3) Most importantly, we hypothesized that resilience and self-efficacy would form a serial mediation pathway, whereby physical activity would positively predict resilience, resilience would positively predict self-efficacy, and self-efficacy would negatively predict anxiety. The proposed conceptual model is depicted in Figure 1. Because the design is cross-sectional, the temporal ordering embodied in these hypotheses represents a theoretical assumption rather than an empirically verifiable causal sequence; the analyses test whether the data are consistent with the proposed ordering, not whether that ordering is correct.

## 2 Method

### 2.1 Participants and procedures

A multi-stage stratified cluster random sampling technique was employed to recruit students from primary, junior high, and senior high schools in the High-tech Zone of Zhuhai City, Guangdong Province, China, with intact classes serving as the sampling units; data were collected between September 2025 and March 2026. Students were eligible if they were currently enrolled at the participating schools and able to complete a Chinese-language questionnaire independently, and questionnaires showing patterned responding or substantial missingness were excluded during data screening, leaving 986 valid cases for analysis. The final sample comprised 483 boys (49.0%) and 503 girls (51.0%), with a mean age of 15.38 years (SD = 1.41); 423 participants (43.0%) attended primary school, 420 (42.8%) junior high school, and 140 (14.2%) senior high school.

To characterize the sample further, participants were grouped by social anxiety severity on the basis of SASC total-score criteria. Ninety-five participants (9.6%) scored below the minimum criterion for the low-anxiety category—that is, they reported minimal social anxiety—and were therefore not assigned to a severity group; the comparisons below accordingly involve 891 participants (low, *n* = 414; middle, *n* = 309; high, *n* = 168), whereas all inferential analyses in this study used the full analytic sample (*N* = 986). The proportion of males differed significantly across anxiety groups (χ^2^ = 6.84, *p* = 0.033), with a lower proportion observed in the high-anxiety group than in the low-anxiety group (43.3% vs. 52.2%, *p* = 0.021). Age also differed significantly across groups (*F* = 14.62, *p* < 0.001): participants in the high-anxiety group were older, on average, than those in the low-anxiety group (15.82 ± 1.47 vs. 15.12 ± 1.35 years, *p* < 0.001). Table 1 presents the demographic characteristics of the sample across anxiety groups.

**Table 1:** Demographic characteristics of the sample across anxiety groups.

Variable	Whole sample	Level			Differences between groups	Comparison between key groups (*p*-value)
		Low anxiety group (*n* = 414)	Middle anxiety group (*n* = 309)	High anxiety group (*n* = 168)		
Gender (male)	483 (49.0%)	240 (52.2%)	125 (49.7%)	31 (43.3%)	χ^2^ = 6.84, *p* = 0.033	Low vs. High: *p* = 0.021
Age (years)	15.38 ± 1.41	15.12 ± 1.35	15.44 ± 1.40	15.82 ± 1.47	*F* = 14.62, *p* < 0.001	Low vs. High: *p* < 0.001
Primary school	423 (43.0%)	182 (44.7%)	76 (18.0%)	75 (17.7%)		Primary school vs. junior high school: 0.008
Junior high school	420 (42.8%)	183 (43.6%)	172 (41.0%)	63 (15.2%)		Middle school vs. high school: 0.112
High school	140 (14.2%)	49 (35.3%)	61 (43.6%)	30 (21.2%)		

Anxiety severity groups were defined by SASC total-score criteria; 95 participants (9.6%) scoring below the low-anxiety criterion were not assigned to a group, so the group ns sum to 891 rather than 986. All inferential analyses reported in this study were based on the full analytic sample (*N* = 986). Percentages for the school-level rows are calculated against the corresponding school-level totals (e.g., *n* = 423 for primary school).

This study received approval from the Ethics Committee of the School of Teacher Education, Northwest Minzu University (approval No. NWNU-EDU-IRB-2023-0316). Written informed consent was obtained from the legal guardians of all participants, and written assent was provided by all child and adolescent participants after they received age-appropriate information about the study procedures and their rights, including the right to withdraw at any time without consequence.

### 2.2 Physical activity

The Chinese version of the Physical Activity Rating Scale (PARS-3) was utilized, consisting of three assessment criteria: exercise intensity, duration of a single session, and frequency of weekly exercise. The PARS-3 was developed originally in Chinese (Liang, 1994), so no translation procedure was required. Each component is rated on a five-level scale, and the total physical activity score is computed with the standard formula: intensity × (duration – 1) × frequency, with intensity and frequency scored 1–5 and duration scored 1–5. Total scores therefore range from 0 to 100, with higher scores indicating a greater volume of physical activity. The PARS-3 has demonstrated adequate reliability and validity in measuring exercise load in Chinese samples (Liang, 1994). In this study, the Cronbach's alpha coefficient for the Physical Activity Scale was 0.774.

### 2.3 Self-efficacy

Self-efficacy was assessed using the Chinese version of the General Self-Efficacy Scale (GSES) (Luszczynska et al., 2005b). The GSES is a unidimensional instrument consisting of 10 items designed to assess an individual's general sense of confidence in dealing with difficulties, setbacks, and unexpected situations. Each item is rated on a 4-point Likert scale ranging from 1 (“not at all true”) to 4 (“exactly true”). Following standard scoring procedures, item scores were summed and divided by 10, with higher mean scores indicating higher levels of general self-efficacy. The Chinese version was developed through standard translation and back-translation procedures and validated in Chinese samples (Wang et al., 2001); it has been widely used in student populations and has demonstrated good psychometric properties, including satisfactory internal consistency (α = 0.87), 1-week test-retest reliability (0.83), and acceptable structural validity. In the present analytic sample (*N* = 986), the GSES showed excellent internal consistency (Cronbach's α = 0.907).

### 2.4 Measurement of mental resilience

This study utilized the Chinese version of the Connor-Davidson Resilience Scale (CD-RISC) (Yu and Zhang, 2007), which includes 25 items designed to assess three aspects of resilience: tenacity, strength, and optimism. The scale employs a five-point Likert system with the following response options: “0 = completely incorrect,” “1 = rarely correct,” “2 = sometimes correct,” “3 = often correct,” and “4 = almost always correct” (Yu and Zhang, 2007). The total score ranges from 0 to 100, with higher scores indicating greater resilience. The Chinese adaptation, produced through translation and back-translation, has been extensively validated for adolescent populations in China, showing excellent reliability and validity (Yu and Zhang, 2007). In the present study, the Cronbach's alpha coefficient was 0.946. A single-construct confirmatory factor analysis (CFA) of the resilience scale in the present sample indicated good fit (χ^2^/df = 1.144, RMSEA = 0.011, CFI = 0.998, TLI = 0.997, IFI = 0.998). Equivalent measurement statistics for all four constructs—composite reliability, average variance extracted, and the range of standardized loadings—were evaluated within the full measurement model and are reported in the Results (Table 2).

**Table 2:** Composite reliability and convergent validity.

Factor	Items	CR	AVE	Min loading	Max loading
Physical activity	4	0.819	0.552	0.349	0.849
Resilience	25	0.945	0.41	0.508	0.735
Self-efficacy	10	0.906	0.49	0.679	0.74
Anxiety	10	0.807	0.296	0.491	0.613

### 2.5 Social anxiety

In this study, the Chinese version of the Social Anxiety Scale for Children (SASC) was used, consisting of 10 items that measure two main aspects: fear of negative evaluation and social avoidance/distress. Participants' responses are rated on a three-point scale: “0 = never,” “1 = sometimes,” and “2 = always.” The total score ranges from 0 to 20, with higher scores reflecting a greater tendency toward social anxiety. The SASC was developed by La Greca et al. (1988), and its Chinese version—produced through standard translation and back-translation—has been validated in Chinese child and adolescent samples, showing good reliability and validity. In this study, the Cronbach's alpha for the scale was 0.806. Because the SASC indexes social anxiety symptoms—fear of negative evaluation and social avoidance and distress—rather than anxiety in a broad transdiagnostic sense, we refer to the outcome as social anxiety throughout the manuscript, and generalization to other anxiety presentations warrants caution.

### 2.6 Statistical analysis

Data were analyzed using structural equation modeling (SEM) in R (version 4.3.1; R Foundation for Statistical Computing, Vienna, Austria) with the lavaan package (Rosseel, 2012). A four-latent-variable serial mediation model was specified, with physical activity as the exogenous latent variable, resilience as the first mediator, self-efficacy as the second mediator, and social anxiety as the endogenous latent outcome. Each latent variable was defined by the individual items of its scale rather than by item parcels or composite indicators; the number of indicators per construct is reported in Table 2. Parameters were estimated with maximum likelihood (ML) estimation, and inference for the indirect effects relied on bias-corrected bootstrap confidence intervals rather than normal-theory standard errors. Before estimation, the data were screened for univariate outliers and multivariate normality: no observation required exclusion, and Mardia's test indicated some degree of multivariate non-normality, which the bootstrap-based inference described below accommodates. To evaluate potential multicollinearity among study constructs, the maximum absolute inter-construct correlation and variance inflation factors (VIFs) for predictors in the structural equations were examined using the analytic sample. VIF values below 5 were interpreted as indicating no problematic multicollinearity. The corresponding diagnostics are reported in the Results. The proportion of missing item responses was below 1%, and Little's test was consistent with a missing-completely-at-random pattern. Missing data were handled using full information maximum likelihood (FIML), which retains all available information under the missing-at-random assumption.

Following common recommendations, χ^2^/df values below 3.00 were considered indicative of acceptable fit, CFI and TLI values above 0.95 indicated excellent fit and values above 0.90 indicated acceptable fit, RMSEA values below 0.06 indicated close fit and values below 0.08 indicated acceptable fit, and SRMR values below 0.08 indicated acceptable fit. Convergent validity was evaluated using standardized factor loadings, composite reliability (CR), and average variance extracted (AVE). Standardized loadings above 0.70 were considered ideal, although values above 0.50 were deemed acceptable; CR values above 0.70 indicated adequate internal consistency; and AVE values above 0.50 indicated sufficient convergent validity. Indirect effects were tested using a bias-corrected bootstrap procedure with 5,000 resamples and 95% confidence intervals. Specifically, the following indirect effects were estimated: the simple indirect effect of physical activity on anxiety through resilience, the simple indirect effect through self-efficacy, the serial indirect effect through resilience and self-efficacy, and the total indirect effect across all mediated pathways. An indirect effect was considered statistically significant when its 95% confidence interval did not include zero. No covariates were included in the primary model; this specification kept the ratio of estimated parameters to observations conservative and preserved comparability with prior serial mediation studies based on the same instruments. We recognize that age, sex, socioeconomic status, and body mass index are plausible confounders of the associations examined here, and we address the implications of their omission in the Limitations section. All statistical tests were two-tailed, and statistical significance was set at *p* < 0.05 (Figure 1).

Two supplementary analyses addressed statistical power and common method bias. No a priori power analysis was conducted when the study was designed; however, the achieved sample (*N* = 986) exceeds conventional benchmarks for models of this size by a comfortable margin, and a *post-hoc* Monte Carlo–based sensitivity analysis indicated power above 0.90 to detect an indirect effect of the magnitude observed for the resilience pathway at α = 0.05. Because all measures were self-reported, common method variance was examined with Harman's single-factor test: the first unrotated factor accounted for 30.1% of the total variance, below the conventional 40% threshold, and a single-factor measurement model fit the data markedly worse than the four-factor model (ΔCFI > 0.20), indicating that method variance is unlikely to account for the pattern of results.

## 3 Results

As shown in Table 3, all four composite scores were based on 986 observations. Resilience showed the highest mean level (M = 3.730, SD = 0.811), followed by physical activity (M = 2.823, SD = 1.136) and self-efficacy (M = 2.570, SD = 0.788), whereas anxiety showed the lowest mean level (M = 1.519, SD = 0.403). Physical activity exhibited the greatest dispersion (SD = 1.136), while anxiety showed the smallest variability (SD = 0.403). In terms of distributional characteristics, physical activity (γ1 = 0.365, γ2 = −1.107) and anxiety (γ1 = 0.956, γ2 = 1.059) were positively skewed, whereas resilience (γ1 = −0.790, γ2 = −0.547) and self-efficacy (γ1 = −0.081, γ2 = −0.956) showed negative skewness. Overall, the descriptive statistics indicated distinct central tendency and dispersion patterns across the four constructs. Absolute values of skewness ( ≤ 0.956) and kurtosis ( ≤ 1.107) fell well within the |2| and |7| bounds commonly taken to justify maximum likelihood estimation, indicating that univariate non-normality posed no serious threat to the structural analyses reported below.

**Table 3:** Descriptive statistics of composite scores.

Construct	*N*	Mean	SD	γ1	γ2
Physical activity	986	2.823	1.136	0.365	−1.107
Resilience	986	3.730	0.811	−0.790	−0.547
Self-efficacy	986	2.570	0.788	−0.081	−0.956
Anxiety	986	1.519	0.403	0.956	1.059

As shown in Table 4, physical activity was significantly and moderately positively correlated with resilience (*r* = 0.533, *p* < 0.001), and significantly and moderately negatively correlated with anxiety (*r* = −0.468, *p* < 0.001). Resilience was also significantly and moderately negatively associated with anxiety (*r* = −0.434, *p* < 0.001). By contrast, self-efficacy was not significantly correlated with physical activity (*r* = 0.024, *p* > 0.05), resilience (*r* = −0.036, *p* > 0.05), or anxiety (*r* = −0.038, *p* > 0.05). The maximum absolute inter-construct correlation was |*r*| = 0.533, which was below conventional thresholds for problematic multicollinearity. Overall, the correlation matrix supported significant associations among physical activity, resilience, and anxiety, whereas self-efficacy showed no meaningful bivariate relationship with the other study variables.

**Table 4:** Pearson correlation matrix.

Variable	Physical activity	Resilience	Self-efficacy	Anxiety
Physical activity	1	0.533^***^	0.024	−0.468^***^
Resilience	0.533^***^	1	−0.036	−0.434^***^
Self-efficacy	0.024	−0.036	1	−0.038
Anxiety	−0.468^***^	−0.434^***^	−0.038	1

^***^p < 0.001.

Additional multicollinearity diagnostics showed no evidence of problematic overlap among predictors. For the anxiety equation, VIF values were 1.401 for physical activity, 1.402 for resilience, and 1.004 for self-efficacy. For the self-efficacy equation, VIF values were 1.398 for both physical activity and resilience. All VIF values were well below the conventional threshold of 5, indicating that multicollinearity was unlikely to bias the structural path estimates.

As shown in Tables 2, 5, the measurement model yielded a chi-square value of 6140.383 with 1,121 degrees of freedom, with a χ^2^/df ratio of 5.478. Model fit was mixed. The absolute indices fell within commonly cited acceptable bounds (RMSEA = 0.064, SRMR = 0.073), whereas the incremental indices remained clearly below the 0.90 criterion for acceptable fit (CFI = 0.805, TLI = 0.796), indicating that the model reproduced the observed covariance structure imperfectly relative to a baseline model. Because the structural portion of the serial mediation model was fully saturated, the SEM necessarily displayed the same fit as the measurement model. With respect to convergent validity, composite reliability was acceptable for all constructs (CRs = 0.807 to 0.945), exceeding the recommended cutoff of 0.70. However, average variance extracted reached the recommended threshold only for physical activity (AVE = 0.552), whereas resilience (AVE = 0.410), self-efficacy (AVE = 0.490), and anxiety (AVE = 0.296) fell below 0.50. Standardized factor loadings ranged from 0.349 to 0.849 for physical activity, 0.508 to 0.735 for resilience, 0.679 to 0.740 for self-efficacy, and 0.491 to 0.613 for anxiety. These values point to limited convergent validity for resilience, self-efficacy, and especially social anxiety, and several indicators loaded weakly (minima of 0.349 and 0.491 for physical activity and anxiety, respectively). We retained all items of the published scales rather than deleting weak indicators *post-hoc*—a choice that preserves construct coverage and cross-study comparability but leaves measurement error in the latent estimates. The structural coefficients reported below should therefore be interpreted as approximate associations, with particular caution for paths involving the anxiety factor.

**Table 5:** CFA and SEM fit indices.

Model	χ^2^	df	χ^2^/df	CFI	TLI	RMSEA	SRMR
CFA	6140.383	1,121	5.478	0.805	0.796	0.064	0.073
SEM	6140.383	1,121	5.478	0.805	0.796	0.064	0.073

The structural path estimates are reported in Table 6. Physical activity was significantly and positively associated with resilience [B = 0.393, SE = 0.026, β = 0.514, *p* < 0.001, 95% CI [0.341, 0.445]]. Physical activity was also significantly and negatively associated with anxiety [B = −0.122, SE = 0.013, β = −0.420, *p* < 0.001, 95% CI [−0.147, −0.096]], and resilience was significantly and negatively associated with anxiety [B = −0.092, SE = 0.015, β = −0.244, *p* < 0.001, 95% CI [−0.122, −0.063]]. By contrast, the paths from physical activity to self-efficacy [B = 0.039, SE = 0.026, β = 0.060, *p* = 0.143, 95% CI [−0.013, 0.090]], from resilience to self-efficacy [B = −0.056, SE = 0.033, β = −0.066, *p* = 0.091, 95% CI [−0.121, 0.009]], and from self-efficacy to anxiety [B = −0.019, SE = 0.014, β = −0.042, *p* = 0.167, 95% CI [−0.046, 0.008]] did not reach statistical significance. Overall, the structural paths supported significant direct associations of physical activity with resilience and anxiety, as well as a significant negative association between resilience and anxiety.

**Table 6:** Structural path coefficients.

Path	B	SE	p	β	BootLLCI	BootULCI
physical_activity → resilience	0.393	0.026	< 0.001	0.514	0.341	0.445
physical_activity → self_efficacy	0.039	0.026	0.143	0.06	−0.013	0.09
resilience → self_efficacy	−0.056	0.033	0.091	−0.066	−0.121	0.009
physical_activity → anxiety	−0.122	0.013	< 0.001	−0.42	−0.147	−0.096
resilience → anxiety	−0.092	0.015	< 0.001	−0.244	−0.122	−0.063
self_efficacy → anxiety	−0.019	0.014	0.167	−0.042	−0.046	0.008

The bootstrap indirect effects are presented in Table 7. Bootstrap analyses indicated that the indirect effect of physical activity on social anxiety through resilience was significant [Indirect 1: estimate = −0.049, 95% CI [−0.064, −0.035]], as the confidence interval did not include zero. By contrast, the indirect effect through self-efficacy alone was not statistically significant [Indirect 2: estimate = −0.001, 95% CI [−0.003, 0]], and the serial indirect effect through resilience and self-efficacy was also not significant [Indirect 3: estimate = 0.001, 95% CI [0, 0.002]], as both confidence intervals included zero. The total indirect effect was significant [estimate = −0.049, 95% CI [−0.064, −0.035]]. In addition, the direct effect remained significant [c′ = −0.113, 95% CI [−0.137, −0.090]], and the total effect was likewise significant [c = −0.162, 95% CI [−0.179, −0.145]]. Overall, the bootstrap results supported a specific indirect effect through resilience, whereas the indirect effect through self-efficacy and the serial mediation pathway were not supported.

**Table 7:** Bootstrap indirect effects.

Effect	Estimate	LLCI	ULCI
Indirect 1: X → M1 → Y	−0.049	−0.064	−0.035
Indirect 2: X → M2 → Y	−0.001	−0.003	0
Indirect 3: X → M1 → M2 → Y	0.001	0	0.002
Total indirect effect	−0.049	−0.064	−0.035
Direct effect (c')	−0.113	−0.137	−0.09
Total effect (c)	−0.162	−0.179	−0.145

## 4 Discussion

The present study examined whether physical activity was associated with lower social anxiety in adolescents through the sequential and independent mediating roles of resilience and self-efficacy. Three findings were central. First, physical activity remained directly and negatively associated with anxiety after the mediators were entered into the model [direct effect, c' = −0.113, 95% CI [−0.137, −0.090]]. Second, the indirect effect through resilience was significant [indirect effect = −0.049, 95% CI [−0.064, −0.035]] and represented the only supported indirect pathway. Third, neither the indirect path through self-efficacy alone [indirect effect = −0.001, 95% CI [−0.003, 0.000]] nor the serial path through resilience and self-efficacy [indirect effect = 0.001, 95% CI [0.000, 0.002]] reached significance. The hypothesized framework was therefore only partially supported: of the three hypothesized indirect pathways, only the one through resilience reached significance. Within the limits of a cross-sectional design and a model with modest incremental fit, the results are consistent with resilience—rather than general self-efficacy—operating as the relevant intervening variable in this sample.

The significant direct path from physical activity to lower anxiety was consistent with the broader literature showing that exercise is associated with meaningful reductions in anxiety symptoms among children and adolescents (Ahn and Fedewa, 2011). Recent high-level syntheses have reported moderate overall anxiolytic effects of exercise in youth, including a meta-meta-analysis of 21 systematic reviews and meta-analyses that found a pooled effect size of SMD = −0.39 for anxiety, and a meta-analysis of randomized trials showing a similar effect (SMD = −0.36), especially when programmes were of sufficient duration, frequency, and adherence to exercise recommendations (Singh et al., 2023). At the same time, some reviews have emphasized that these benefits are heterogeneous and sometimes modest, particularly across different populations and intervention formats (Biddle et al., 2019). Against that background, the persistence of a significant direct effect in the present study suggested that the psychological mechanisms included here did not exhaust the ways in which physical activity may relate to lower anxiety (Lubans et al., 2016). Several complementary processes, none captured by our model, plausibly contribute to the remaining direct association. Exercise modulates hypothalamic–pituitary–adrenal axis reactivity and autonomic balance, improves sleep, and lifts affective tone, and it typically embeds adolescents in structured peer interaction; each of these processes has been linked to lower anxiety in mechanistic reviews (Lubans et al., 2016; Gerber and Pühse, 2009). Cognitive-behavioral dimensions of activity engagement may matter as well, as suggested by evidence that cognitively and behaviorally regulated physical activity relates to adolescent wellbeing (Derelioglu et al., 2025). We list these as candidate mediators for future models, not as explanations established by the present data.

The strongest and only significant indirect pathway operated through resilience, which provided the clearest explanatory candidate in the current data (Luo et al., 2025). This finding was theoretically coherent with resilience theory and the stress-buffering model, both of which suggest that repeated exposure to manageable challenge, recovery, and adaptive coping strengthens psychological resources that protect against distress (Masten, 2018). Physical activity may foster resilience by placing adolescents in situations that require persistence, frustration tolerance, emotional regulation, goal adjustment, and recovery from temporary failure (Eime et al., 2013). These repeated experiences can cultivate a more adaptive response repertoire when stressors arise outside sport or exercise settings (Gerber and Pühse, 2009). Empirically, this interpretation aligns with a recent meta-analysis showing a reliable positive association between physical activity and resilience in young students, as well as with longitudinal work indicating that physical activity predicts later resilience and that resilience, in turn, predicts lower subsequent social anxiety (Wu et al., 2025; Yarrington et al., 2022). In this sense, resilience appears to function as a relatively proximal protective resource through which physically active adolescents may become less vulnerable to anxiety symptoms (Fritz et al., 2018). Two caveats temper this interpretation. The AVE for resilience (0.410) fell below the 0.50 benchmark, so the latent resilience factor carried non-trivial measurement error; and the model's incremental fit was modest, which counsels against strong mechanistic claims. The resilience pathway is accordingly best read as suggestive—consistent with, but not demonstrative of, a protective process—pending longitudinal replication.

By contrast, the non-significant self-efficacy findings require more careful interpretation (Zhao et al., 2010). Contrary to the hypothesized model, physical activity did not significantly predict self-efficacy, resilience did not significantly predict self-efficacy, and self-efficacy did not significantly predict anxiety (Dishman et al., 2004). One reading of this pattern is that general self-efficacy, as operationalized here, was too distal from the constructs at either end of the model—particularly because the anxiety measure indexed social anxiety symptoms rather than anxiety in a broad transdiagnostic sense (Muris, 2002). This explanation is consistent with social cognitive theory, which conceptualizes self-efficacy as domain- and task-specific rather than as a uniformly general trait (Bandura, 1997). Recent adolescent studies that did find significant mediation typically focused on sport-specific or emotional/social self-efficacy—constructs that are more proximal both to physical activity participation and to social-evaluative fears (Tang et al., 2022). For example, sports self-efficacy has been shown to mediate the association between physical exercise and adolescent social anxiety, and emotional/social self-efficacy has also been linked to anxiety trajectories in prevention research (Bandura et al., 2003). In addition, recent evidence has suggested that resilience can partially transmit the association between physical exercise and self-efficacy (Peng et al., 2025). Construct breadth is, however, only one of several plausible explanations, and the present data cannot adjudicate among them. Psychometric attenuation offers a second: the AVE values for self-efficacy (0.490) and especially social anxiety (0.296) imply substantial measurement error, which biases structural paths toward zero. A third possibility is genuinely weak coupling in this developmental period, since general self-efficacy showed no meaningful bivariate association with any study variable (|*r*| ≤ 0.038)—a pattern difficult to attribute to modeling choices alone (Luszczynska et al., 2005a). Each account predicts the same null result here, and only designs with domain-matched measures and stronger indicators can separate them.

This combination of supported and unsupported pathways places the present findings in a nuanced position relative to previous work (Zhao et al., 2010). On the one hand, the results converged with a large body of evidence showing that physical activity is inversely associated with anxiety and that resilience is an important protective factor in adolescent mental health (Li et al., 2023). On the other hand, the results diverged from studies reporting that self-efficacy mediates the relationship between physical activity and mental health or social anxiety in adolescents. The divergence becomes intelligible once key design differences are made explicit. Leng et al. (2025) observed significant mediation through general self-efficacy in Chinese adolescents, but their outcome was academic stress—a domain in which generalized competence beliefs are directly implicated—and their chain placed self-efficacy before resilience. Tang et al. (2022) reported efficacy-mediated pathways using emotion regulation self-efficacy in college students rather than school-aged adolescents. Li et al. (2024) did find serial mediation to social anxiety, yet through resilience and sleep problems, and again in a college sample. Relative to these studies, the present design paired a general efficacy measure with a socially specific outcome in a younger sample—precisely the configuration in which social cognitive theory would predict the weakest coupling (Bandura, 1997). The discrepancy is informative rather than problematic (Maddux and Kleiman, 2021). It suggests that the role of self-efficacy may depend on how the construct is operationalized, which mental health outcome is studied, and whether the context of the efficacy belief matches the context of the behavioral exposure and the symptom domain (Maddux and Kleiman, 2021). This interpretation is also consistent with recent reviews showing that the mental health effects of physical activity in healthy adolescents are not entirely uniform and may vary according to intervention design, outcome definition, and measurement precision (Ruiz-Ranz and Asín-Izquierdo, 2025).

The theoretical implications should be stated with restraint. The study set out to test a serial mediation framework; the data supported a simpler structure. Physical activity related to lower social anxiety directly and through resilience, while every path involving general self-efficacy was non-significant. Rather than claiming refinement of the full chain, we draw two narrower conclusions. Resilience behaved as the framework predicted and merits a central place in models linking adolescent physical activity to social anxiety—a conclusion reinforced by longitudinal evidence in comparable samples (Wu et al., 2025). Efficacy beliefs, by contrast, appear to contribute only when their domain matches the outcome under study: social or sports-related efficacy for social-evaluative symptoms, for instance. Future models should therefore treat construct–outcome alignment as a substantive specification decision rather than a measurement afterthought.

## 5 Limitations

Several limitations should be acknowledged. First, the cross-sectional design precluded causal inference. Although the SEM tested directional pathways, the temporal ordering remained theoretical rather than demonstrated; future longitudinal, cross-lagged, or randomized intervention studies are needed to verify whether changes in physical activity precede changes in resilience and anxiety. Second, all variables were assessed via self-report, which raised the possibility of recall bias, social desirability bias, and shared method variance. Future work should combine questionnaire data with objective indicators of physical activity, such as accelerometry, and, where possible, include parent, teacher, or clinician ratings of anxiety-related functioning. Third, the measurement model itself warrants caution. In the present data, RMSEA and SRMR suggested acceptable absolute fit, but CFI and TLI were below conventional thresholds, and AVE values were suboptimal for resilience, self-efficacy, and especially anxiety. These psychometric features mean that the latent estimates should be interpreted cautiously and that future research should consider refining item parcels, testing alternative measurement structures, or using more domain-matched constructs. Fourth, the self-efficacy measure captured general rather than social or sports-related efficacy, which may have attenuated the hypothesized pathway. Fifth, the sample consisted of Chinese adolescents, and the anxiety outcome was operationalized primarily as social anxiety symptoms; accordingly, generalizability to other cultural contexts, age groups, or clinical anxiety presentations remains uncertain. Finally, no covariates were included in the primary model, and the omission of potential confounders deserves emphasis. Variables such as sex, age, socioeconomic status, sleep, and body mass index plausibly relate to both physical activity and social anxiety; to the extent that they do, their exclusion will have inflated the estimated associations, whereas confounders acting in opposite directions on predictor and outcome would produce the reverse bias. Because these variables were not modeled, the net direction of bias cannot be determined, and the reported coefficients are best treated as unadjusted associations. Sex and age may additionally moderate, rather than merely confound, the pathways examined. Future studies should account for potentially important confounders and moderators, including sex, age, socioeconomic status, sleep, BMI, family climate, social support, exercise type, exercise intensity, and prior mental health status, and should also test other plausible mediators such as emotion regulation, body image, self-esteem, and neurobiological stress markers.

## 6 Conclusion

Two conclusions follow from this study, and both carry the caution appropriate to cross-sectional evidence. In a large sample of Chinese adolescents, physical activity related to lower social anxiety directly and, more modestly, through higher resilience. The second hypothesized resource, general self-efficacy, played no detectable role—neither as an independent mediator nor as a link in the proposed serial chain—so the data favor a parsimonious activity–resilience–social anxiety structure over the full serial mediation model.

These findings suggest several concrete priorities. For practice, programmes that simply increase activity volume may capture only part of the potential benefit; embedding deliberate resilience-building elements—graded challenge, coping rehearsal, structured recovery from setbacks—within school physical activity offers a testable route to larger effects (Eime et al., 2013). For measurement, researchers studying social anxiety outcomes should prefer social or exercise-specific efficacy scales over general ones and should attend to the weak convergent validity observed here for brief anxiety measures in this age group. For inference, the resilience pathway now needs longitudinal and experimental scrutiny: cross-lagged panel designs can probe the assumed temporal ordering, and cluster-randomized activity interventions measuring resilience as a process variable can establish whether the pathway is causal. Until such evidence accumulates, the serial mediation framework should be treated as an open hypothesis rather than an established account.

## Data Availability

The original contributions presented in the study are included in the article/supplementary material, further inquiries can be directed to the corresponding authors.
